# Social support and physical activity: does general health matter?

**DOI:** 10.1186/s11556-024-00347-6

**Published:** 2024-06-20

**Authors:** Sarah B. Lieber, Jerad Moxley, Lisa A. Mandl, M. Carrington Reid, Sara J. Czaja

**Affiliations:** 1 Division of Rheumatology, Hospital for Special Surgery, 535 East 70th Street, New York, NY USA; 2 Department of Medicine, Weill Cornell Medicine, 530 East 70th Street, New York, NY USA; 3 Division of Geriatrics and Palliative Medicine, Weill Cornell Medicine, 1300 York Avenue, New York, NY USA

**Keywords:** Social support, General health, Physical activity, Older adults

## Abstract

**Background:**

Physical activity levels remain suboptimal in older adults. Exploration of potentially modifiable factors such as social support is needed to inform the development and implementation of patient-oriented physical activity interventions for older adults. The impact of general health on the relationship between social support and physical activity is not well understood. We aimed to determine the association between social support and self-reported physical activity in a study of community-dwelling older adults. In addition, we examined whether self-reported general health mediates the relationship between social support and self-reported physical activity.

**Method:**

This cross-sectional study analyzed baseline data collected as part of a randomized controlled trial comparing a digital physical activity intervention, which included social support features, with a tablet-based educational control. Adults ≥ 60 years of age were enrolled at 2 sites. Self-reported general health, social support, physical activity, and sociodemographic characteristics and comorbid conditions were assessed. Pearson and point-biserial correlations were computed to evaluate the relationship between physical activity and general health, social support, and sociodemographic features. Social support (exposure), general health (mediator), and physical activity (outcome) were incorporated into a mediation model.

**Results:**

Among 181 participants (mean age of 70.1 years), significant correlations were found between physical activity and both general health and social support (*r* = -0.19 and *r* = 0.21, respectively; both *p* < 0.01). General health significantly mediated the relationship between social support and physical activity (unstandardized ß coefficient 416.9; 95% confidence interval 96.4, 842.0).

**Conclusions:**

Augmentation of social support, particularly when coupled with other modes of health promotion to improve personal wellbeing, may be a valuable component of physical activity promotion programs. Further longitudinal research is needed to clarify the potential mechanistic pathways linking social support, general health, and physical activity to inform development of evidence-based physical activity interventions for older adults and improve downstream health-related outcomes.

**Trial Registration:**

ClinicalTrials.gov, ClinicalTrials.gov identifier NCT03538158. Registered May 25, 2018.

## Background

Benefits of physical activity are well established in older adults, including reduced risk of cardiovascular disease, cerebrovascular disease, diabetes mellitus, some forms of cancer, as well as enhanced mental health and quality of life [1]. In recognition of these benefits, the United States Centers for Disease Control and the World Health Organization advise ≥ 150 min of moderate-intensity physical activity or 75 min of vigorous-intensity physical activity per week, along with muscle strengthening activities ≥ 2 days per week and balance improvement activities 3 days per week for adults ≥ 65 years of age [2, 3]. Nevertheless, most studies report low rates of adherence to physical activity guideline recommendations among community-dwelling older adults [4]. For instance, in a recent cross-sectional study of participants ≥ 65 years of age in the National Health and Nutrition Examination Survey in the 2017–2018 wave, only 34.2% (95% confidence interval 30.2–38.3%) were found to meet physical activity guideline recommendations [5]. Older old adults, defined as ≥ 80 or 85 years of age, are especially prone to physical inactivity [4]. There may be variability in these adherence estimates due to use of different validated physical activity instruments, as well as differential age and geographic distributions among observational studies [4].

Considerable evidence supports the use of exercise interventions in the general population of older adults to enhance physical activity levels and improve downstream health-related outcomes [6, 7]. Exercise interventions incorporating resistance training, meditative movements, and active videogames, have been found to be particularly effective [7]. Nevertheless, optimal programming, including mode of delivery, intensity and duration of exercise, and accompanying features, has not been well defined [6, 7]. While multiple determinants of physical activity behavior in older adults, including age, gender, and walkability, have been identified, little is known about other potentially modifiable factors impacting physical activity behavior in older adults [8]. Exploration of additional factors is needed to inform development of physical activity interventions for older adults that will lead to durable treatment effects.

The positive influence of social support on physical activity is well studied. Social support has been found to promote self-efficacy, which in turn encourages physical activity [9, 10]. Older individuals with greater physical activity-related social support, particularly from family members, are generally more likely to engage in physical activity [11]. However, the relationship between overall social support (i.e., not specifically physical activity-related) and engagement in physical activity is less clear [11]. For example, in a 2017 systematic review of social support and physical activity among adults ≥ 60 years of age (including 22 cross-sectional, 3 prospective/longitudinal, and 2 interventional studies) physical activity-related social support was associated with increased levels of physical activity in 11 of 17 studies (65%) [11]. Only 4 studies (including 5 analyses), assessed the association between overall social support or engagement and physical activity, with 2 (40%) demonstrating a significant association [12–15]. In a 2022 systematic review of reviews on barriers and facilitators of physical activity in adults across the lifespan, higher levels of overall social support were associated with more leisure-time physical activity in 12 of 17 studies [16]. Other factors, such as general health, which could mediate the relationship between overall social support and physical activity in older adults have not been investigated as extensively.

Greater levels of self-reported general health have been associated with higher self-reported physical activity levels in multiple cross-sectional studies of the general adult population [17]. However, to our knowledge, limited attention has been devoted to disentangling the relationship of social support, self-reported general health, and engagement in physical activity. In this exploratory study, we aimed to determine the cross-sectional association between overall social support and self-reported physical activity in a sample of community-dwelling older adults. In addition, we examined whether self-reported general health mediates the relationship between overall social support and self-reported physical activity.

## Methods

This study analyzed cross-sectional data collected as part of a randomized controlled trial that compared the Fittle Senior System (FSS), a digital physical activity intervention with social support features drawing on social cognitive theory [9, 18], with a digital educational control. The study protocol is summarized briefly below.

### Protocol

Participants were enrolled at two academic medical centers in the United States. Following telephone pre-screening and informed consent, participants were assigned randomly in blocks of 3–6 individuals to receive either the FSS or an educational control. FSS arm participants received a tablet delivering pictorial and written instructions on physical exercises over a 12-week period, with team-based social support features in the form of 1-to-1 and group chat functions. Participants randomized to the educational control received a tablet preloaded with widely available content on physical exercise and safety tips.

### Sample

Eligible participants were ≥ 60 years of age and able to speak English, read at the sixth-grade level, and pass the Telephone Interview for Cognitive Status [19]. Individuals with cognitive (defined by score < 26 on the Mini Mental Status Exam [20]) or visual (corrected or uncorrected visual acuity < 20/40) impairment, active participation in a structured physical exercise regimen, or health conditions that could affect their ability to participate were excluded. Recruitment was conducted through classified advertisements, virtual flyers, community newsletters distributed at senior centers and in geriatric clinics, virtual presentations, and social media advertisements.

### Measures

Multiple measures were collected as part of the parent randomized controlled trial. Measures relevant to the current study are described below.

#### General health

General health was measured using the first question of the 36-Item Short-Form Survey (SF-36): “In general would you say your health is…?” Scores range from 1 (“excellent”) to 5 (“poor”) [21]. Due to use of a single measure, Cronbach’s alpha for general health could not be calculated.

#### Social support

Social support was measured using the 12-Item Interpersonal Support Evaluation List [22]. According to this scale, social support is measured across 3 subscales: appraisal, belonging, and tangible. Each item is scored on a 1 (“definitely false”) to 4 (“definitely true”) scale, including reverse coding for some prespecified items. We have reported the mean score, with a higher mean corresponding with more social support. In our sample, Cronbach’s alpha for social support was 0.87.

#### Physical activity

Physical activity was measured using the Global Physical Activity Questionnaire [23]. This 16-item scale assesses physical activity across multiple domains, including activity performed at work, during travel to and from places, and recreational activities, as well as sedentary behavior. Physical activities are clustered into moderate and vigorous physical activities, and physical activity levels are expressed as the total number of metabolic equivalent (met)-minutes per week. Attainment of  ≥ 150 minutes of moderate-intensity or 75 min of vigorous-intensity physical activity per week or ≥ 600 metabolic equivalent-minutes per week of moderate and vigorous physical activity is consistent with adherence to United States Centers for Disease Control and the World Health Organization physical activity guidelines [2, 3]. Sedentary behavior based on the Global Physical Activity Questionnaire is expressed in terms of minutes per week [23]. World Health Organization physical activity guideline recommendations advise limiting sedentary activities without defining a specific goal threshold [3]. In a recent systematic review, reliability and validity of the Global Physical Activity Questionnaire were found to vary based on study population [24]. In our sample, Cronbach’s alpha for physical activity was 0.51.

#### Sociodemographic characteristics

Sociodemographic and related features, including age, gender, race, ethnicity, and comorbid conditions, were collected by self-report.

### Analysis

**Fig. 1:**
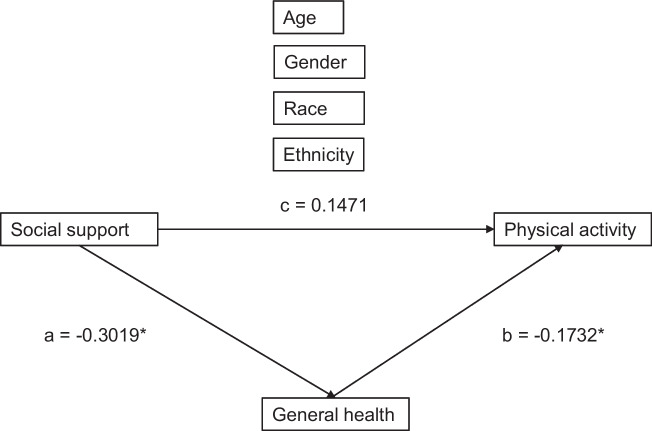
Mediation model relating social support and self-reported general health to self-reported physical activity. Parameter displayed is the standardized beta coefficient. Asterix reflects a statistically significant path based on bootstrap confidence interval

Sample characteristics were summarized using descriptive statistics. Participants without complete data were excluded from subsequent analyses. Pearson and point-biserial correlations were computed to evaluate the relationship between self-reported physical activity and self-reported general health, social support, and sociodemographic and related features. For the main analysis, we conducted a mediation analysis with bootstrap confidence intervals. We incorporated social support (exposure), self-reported general health (mediator), self-reported physical activity (outcome), and the sociodemographic features of age, gender, and race and ethnicity (covariates) into the mediation model determined a priori based on review of the literature [11]. Figure 1 shows the mediation model. For model estimation, 5000 bootstrap samples were employed to attain 95% confidence intervals, which were deemed significant if they did not overlap with 0. SPSS Statistics 29 was used for statistical analysis.

## Results

### Sample characteristics

From 2018–2021, 471 potential participants were screened, and 228 were found initially to be eligible for randomization; 34 eligible individuals did not proceed to randomization: 12 were found to be ineligible on further assessment, 24 were lost to contact, 9 withdrew, and 1 was not randomized for other reasons. Baseline data were available for 181 randomized participants.

Sample characteristics are presented in Table 1. Participants had mean age of 70.1 years, were 80.1% women, with 13.4% self-identifying as Black or African American and 12.7% as Hispanic or Latino. Arthritis and hypertension were the most common self-reported comorbid conditions, present in 55.6% and 39.7%, respectively. Participants’ mean general health and social support scores were 2.4 [(standard deviation [SD] 0.8; range 1 (“excellent”) to 5 (“poor”)] and 3.1 (SD 0.6; range 0–4; higher score indicates more social support), respectively. Mean self-reported physical activity level was 1997.2 (SD 4538.7) metabolic equivalent-minutes/week while median self-reported physical activity level was 720.0 (interquartile range 0, 2055.0) metabolic equivalent-minutes/week. Mean self-reported sedentary activity was 406.9 (SD 204.9) minutes/week. Among 3 participants for whom complete baseline data were unavailable, mean age was 67.3 years, 2 were women, and 1 self-identified as Black or African American.

**Table 1:** Participant characteristics

Domain	*N* = 181	
	N (%)	Mean (standard deviation)
Age (years)		70.1 (6.8)
Gender		
Female	145 (80.1)	
Race		
Black or African American	24 (13.4)	
White	146 (80.7)	
Other	11 (6.1)	
Ethnicity		
Hispanic or Latino	23 (12.7)	
Self-reported comorbid conditions		
Arthritis	99 (55.6)	
Bronchitis	47 (26.9)	
Malignancy	31 (17.8)	
Diabetes mellitus	28 (15.8)	
Heart disease	17 (9.7)	
Hypertension	69 (39.7)	
Stroke	12 (6.9)	
Self-reported general health^a^		2.4 (0.8)
Social support^b^		3.1 (0.6)
Self-reported physical activity^c^ (metabolic equivalent-minutes/week)		1997.2 (4538.7)
Sedentary activity^c^ (minutes/week)		406.9 (204.9)

^a^First question of 36-Item Short Form Survey: Ranges from 1 (“excellent”) to 5 (“poor”)

^b^12-Item Interpersonal Support Evaluation List: Ranges from 0–4; higher score indicates more social support

^c^Global Physical Activity Questionnaire

### Correlations between physical activity and sociodemographic variables, general health, and social support

Pearson and point-biserial correlations between self-reported physical activity and sociodemographic variables, self-reported general health, and social support are displayed in Table 2. Complete data were available for analysis on 178 participants. Greater self-reported general health (*r* = -0.19, *p* < 0.01) and greater social support (*r* = 0.21, *p* < 0.01) were significantly correlated with greater self-reported physical activity levels.

**Table 2:** Pearson and point-biserial correlations between self-reported physical activity^a^ and sociodemographic variables, self-reported general health^b^, and social support^c^ (*N* = 178)

Domain	r	*p*
Age	-0.106	0.08
Gender	0.045	0.28
Race		
Black or African American	0.215	< 0.01
Other	-0.041	0.29
Ethnicity		
Hispanic or Latino	-0.061	0.21
General health^b^	-0.188	< 0.01
Social support^c^	0.205	< 0.01

^a^Global Physical Activity Questionnaire

^b^36-Item Short Form Survey General Health Scale

^c^12-Item Interpersonal Support Evaluation List

### Mediation Model

Table 3 shows the mediation model relating social support and self-reported physical activity, as mediated by self-reported general health, with age, gender, and race and ethnicity added to the model as covariates.

**Table 3:** Mediation model relating social support^a^ and self-reported general health^b^ to self-reported physical activity^c^ (*N* = 178)

Model domain	Standardized ß coefficient	Unstandardized ß coefficient [95% confidence interval]
Direct effects on general health		
Age	-0.19	-0.02 [-0.04, -0.01]
Gender	< 0.01	0.01 [-0.27, 0.28]
Race and ethnicity		
Black or African American	0.07	0.17 [-0.17, 0.50]
Other	0.11	0.44 [-0.16, 1.03]
Hispanic or Latino	0.11	0.25 [-0.07, 0.58]
Social support	-0.30	-0.40 [-0.59, -0.21]
Direct effects on physical activity		
Age	-0.08	-51.00 [-152.40, 50.39]
Gender	0.02	172.51 [-1510.66, 1855.69]
Race and ethnicity		
Black or African American	0.19	2646.05 [581.04, 4711.06]
Other	-0.02	-462.44 [-4113.77, 3188.89]
Hispanic or Latino	-0.05	-696.91 [-2701.84, 1308.02]
General health	-0.17	-1051.13 [-1974.03, -128.24]
Social support	0.15	1172.83 [-54.48, 2400.13]
Total effect of social support on physical activity	0.20	1589.7 [404.5, 2774.9]
Indirect effect of social support on physical activity mediated by general health	0.05	416.9 [96.4, 842.0]

^a^12-Item Interpersonal Support Evaluation List

^b^36-Item Short Form Survey General Health Scale

^c^Global Physical Activity Questionnaire

Social support had a significant direct effect on self-reported general health (unstandardized ß = -0.40, 95% CI [-0.59, -0.21]) (see Path a in Fig. 1 for the standardized coefficient). Self-reported general health had a significant direct effect on self-reported physical activity (unstandardized ß = -1051.13, 95% CI [-1974.03, -128.24]) (see Path b in Fig. 1 for the standardized coefficient). However, social support was not significantly associated with self-reported physical activity (unstandardized ß = 1172.83, 95% CI [-54.48, 2400.13]) (see Path c in Fig. 1 for the standardized coefficient). Most importantly, self-reported general health significantly mediated the effect of social support on self-reported physical activity as the indirect effect of self-reported general health on self-reported physical activity was significant (unstandardized ß = 416.9, 95% CI [96.4, 842.0]). General health accounted for 49.58% of the effect of social support on physical activity after accounting for the effect of covariates.

## Discussion

In this exploratory study, we examined the association between overall social support and self-reported physical activity, as well as the role of general health as a mediator of this relationship in a sample of community-dwelling older adults. We found a significant association between overall social support and physical activity. However, we also found a significant mediating effect of general health on the relationship between overall social support and physical activity such that after controlling for self-reported general health, social support was no longer significantly associated with physical activity.

Our findings add to existing evidence on the association of social support with physical activity in older adults. In corroboration with some, but not all prior studies, we found a significant association between overall social support based on the 12-item Interpersonal Social Evaluation List and self-reported physical activity [22]. Heterogeneity in study design and modes of assessment of overall social support and physical activity levels may contribute to the observed differences among study findings. Additional studies in larger samples of community-dwelling older adults are needed to clarify the association between overall social support and physical activity. Evaluation of the relationship of specific aspects of social support with physical activity is also warranted as this will identify the type of social support that has the most impact on engagement in physical activity, which will inform future interventions.

Our findings highlight general health as a significant mediator of the relationship between overall social support and physical activity in older adults. We hypothesize that an individual’s perceived general health status may impact both their ability to seek and/or accept social support and their ability and/or motivation to engage in physical activity. As social support is known to be protective against depression in older adults [25], enhanced social support may lead to enhanced perception of general health by reducing depression, thereby facilitating engagement in physical activity. Further, as social support may be associated with physician utilization [26], greater social support may improve general health by encouraging routine ambulatory care visits, creating opportunities for counseling regarding the benefits of a physically active lifestyle. Our findings suggest that perceived general health should be considered in interventional studies aimed at improving physical activity via social support enhancement strategies. This may be particularly important in older adults with chronic conditions, who tend to have worse perceived health [27].

Our study has several limitations. The cross-sectional nature of this analysis precludes causal inferences, though the directionality of the mediation model is rooted in the existing literature [11]. Participants were enrolled at two sites in the United States, potentially limiting generalizability of our findings to individuals from other countries or regions. Our study was conducted during the height of the Covid-19 pandemic, which likely impacted social support [28] and physical activity levels [29]. Further, participants were, on average, physically active by self-report; the extent to which our results can be extended to physically inactive individuals is not clear. Finally, we used a self-reported measure of physical activity, which is known to be incompletely concordant with objective physical activity levels and often over-reported [30]. Collection of objective measures of physical activity was not feasible as this study was conducted at the height of the Covid-19 pandemic.

## Conclusions

Our results highlight individual perception of general health as an important mediator of the relationship between social support and physical activity, suggesting that attention to general health status is needed to optimize the potential benefits of social support in the context of physical activity promotion. Future research incorporating longitudinal designs and involving racially and ethnically diverse older adults is needed to clarify the potential mechanistic pathways linking social support, general health, and physical activity to inform development of evidence-based physical activity interventions for older adults and thereby improve downstream health-related outcomes.

## Data Availability

The data underlying this article cannot be shared publicly due to the privacy of individuals who participated in the study. De-identified data will be shared on reasonable request to the corresponding author after Institutional Review Board approval and completion of a data use agreement.

## Abbreviations

FSS: Fittle Senior System
SF-36: 36-Item Short-Form Survey
SD: Standard deviation
CI: Confidence interval
